# Supplementary material for: Description of Bothriurus mistral n. sp., the highest-dwelling Bothriurus from the western Andes (Scorpiones, Bothriuridae), using multiple morphometric approaches
Source: PLoS One. 2023 Feb 22;18(2):e0281336. doi: 10.1371/journal.pone.0281336 (PMC9946213; doi:10.1371/journal.pone.0281336)

PLOS ONE

Description of Bothriurus mistral n. sp., the highest-dwelling Bothriurus from the western Andes (Scorpiones, Bothriuridae), using multiple morphometric approaches

--Manuscript Draft--

**Manuscript Number:**	PONE-D-22-27368
**Article Type:**	Research Article
**Full Title:**	Description of Bothriurus mistral n. sp., the highest-dwelling Bothriurus from the western Andes (Scorpiones, Bothriuridae), using multiple morphometric approaches
**Short Title:**	Description of the high Andean species Bothriurus mistral n. sp.
**Corresponding Author:**	Andrés Alejandro Ojanguren-Affilastro, Doctor<br>Museo Argentino de Ciencias Naturales Bernardino Rivadavia<br>Ciudad Autonoma de Buenos Aires, Ciudad Autonoma de Buenos Aires ARGENTINA
**Keywords:**	Scorpiones; Andes; Bothriurus; new species; Morphometrics; high altitudes; Chile
**Abstract:**	We describe Bothriurus mistral n. sp.(Scorpiones, Bothriuridae) from the Chilean north-central Andes of the Coquimbo Region. This is the highest elevational discovery for Bothriurus in the western slopes of the Andes. This species was collected in the Estero Derecho Private Protected Area and Natural Sanctuary as part of the First National Biodiversity Inventory of Chile of the Integrated System for Monitoring and Evaluation of Native Forest Ecosystems (SIMEF). Bothriurus mistral n. sp. is closely related to Bothriurus coriaceus Pocock, 1893, from the lowlands of central Chile. This integrative research includes a combination of traditional morphometrics and geometric morphometric analyses to support the taxonomic delimitation of the species.
**Order of Authors:**	Andrés Alejandro Ojanguren-Affilastro, Doctor
	Hugo A. Benitez, Dr.
	Hernán Augusto Iuri, Licenciado
	Camilo Iván Mattoni, Dr.
	Fermin Alfaro, Dr.
	Jaime Pizarro-Araya, Dr.
**Additional Information:**	
**Question**	**Response**
**Financial Disclosure**<br><br>Enter a financial disclosure statement that describes the sources of funding for the work included in this submission. Review the submission guidelines for detailed requirements. View published research articles from *PLOS ONE* for specific examples.<br><br>This statement is required for submission and **will appear in the published article** if the submission is accepted. Please make sure it is accurate.	DIDULS PR2121210 and DIDULS PEQMEN212124 projects of the University of La Serena, Chile, and the funding from the Ministry of Education of Chile, through MINEDUC's performance agreement: Implementation of a competitive model of innovation and creation: preparing the University of La Serena for 2030, ULS19101, ANID FB210006 grant, and the SIMEF project (INFOR-IEB Agreement) to JPA.<br>PICT 2019-597 project by Agencia Nacional de Promoción Científica y Tecnológica (Argentina) to AAOA.

Powered by Editorial Manager® and ProduXion Manager® from Aries Systems Corporation

**Unfunded studies**<br>Enter: *The author(s) received no specific funding for this work.*<br><br>**Funded studies**<br>Enter a statement with the following details:<br>• Initials of the authors who received each award<br>• Grant numbers awarded to each author<br>• The full name of each funder<br>• URL of each funder website<br>• Did the sponsors or funders play any role in the study design, data collection and analysis, decision to publish, or preparation of the manuscript?<br>• **NO** - Include this sentence at the end of your statement: *The funders had no role in study design, data collection and analysis, decision to publish, or preparation of the manuscript.*<br>• **YES** - Specify the role(s) played.<br><br>* typeset	
**Competing Interests**<br><br>Use the instructions below to enter a competing interest statement for this submission. On behalf of all authors, disclose any competing interests that could be perceived to bias this work—acknowledging all financial support and any other relevant financial or non-financial competing interests.<br><br>This statement is required for submission and **will appear in the published article** if the submission is accepted. Please make sure it is accurate and that any funding sources listed in your Funding Information later in the submission form are also declared in your Financial Disclosure statement.<br><br>View published research articles from *PLOS ONE* for specific examples.	The authors have declared that no competing interests exist.

**NO authors have competing interests**<br><br>Enter: *The authors have declared that no competing interests exist.*<br><br>**Authors with competing interests**<br><br>Enter competing interest details beginning with this statement:<br><br>*I have read the journal's policy and the authors of this manuscript have the following competing interests: [insert competing interests here]*<br><br>* typeset	
**Ethics Statement**<br><br>Enter an ethics statement for this submission. This statement is required if the study involved:<br><br>• Human participants<br>• Human specimens or tissue<br>• Vertebrate animals or cephalopods<br>• Vertebrate embryos or tissues<br>• Field research<br><br>Write "N/A" if the submission does not require an ethics statement.<br><br>General guidance is provided below. Consult the submission guidelines for detailed instructions. **Make sure that all information entered here is included in the Methods section of the manuscript.**	Collection permits of the local community managing the Private Protected Area and Natural Sanctuary of Estero Derecho were obtained.

Powered by Editorial Manager® and ProduXion Manager® from Aries Systems Corporation

**Format for specific study types**<br><br>**Human Subject Research (involving human participants and/or tissue)**<br>• Give the name of the institutional review board or ethics committee that approved the study<br>• Include the approval number and/or a statement indicating approval of this research<br>• Indicate the form of consent obtained (written/oral) or the reason that consent was not obtained (e.g. the data were analyzed anonymously)<br><br>**Animal Research (involving vertebrate animals, embryos or tissues)**<br>• Provide the name of the Institutional Animal Care and Use Committee (IACUC) or other relevant ethics board that reviewed the study protocol, and indicate whether they approved this research or granted a formal waiver of ethical approval<br>• Include an approval number if one was obtained<br>• If the study involved *non-human primates*, add *additional details* about animal welfare and steps taken to ameliorate suffering<br>• If anesthesia, euthanasia, or any kind of animal sacrifice is part of the study, include briefly which substances and/or methods were applied<br><br>**Field Research**<br><br>Include the following details if this study involves the collection of plant, animal, or other materials from a natural setting:<br>• Field permit number<br>• Name of the institution or relevant body that granted permission	
**Data Availability**<br><br>Authors are required to make all data underlying the findings described fully available, without restriction, and from the time of publication. PLOS allows rare exceptions to address legal and ethical concerns. See the PLOS Data Policy and FAQ for detailed information.	Yes - all data are fully available without restriction

A Data Availability Statement describing where the data can be found is required at submission. Your answers to this question constitute the Data Availability Statement and **will be published in the article**, if accepted.<br><br>**Important:** Stating 'data available on request from the author' is not sufficient. If your data are only available upon request, select 'No' for the first question and explain your exceptional situation in the text box.<br><br>Do the authors confirm that all data underlying the findings described in their manuscript are fully available without restriction?	
**Describe where the data may be found in full sentences. If you are copying our sample text, replace any instances of XXX with the appropriate details.**<br><br>• If the data are **held or will be held in a public repository**, include URLs, accession numbers or DOIs. If this information will only be available after acceptance, indicate this by ticking the box below. For example: *All XXX files are available from the XXX database (accession number(s) XXX, XXX.*).<br>• If the data are all contained **within the manuscript and/or Supporting Information files**, enter the following: *All relevant data are within the manuscript and its Supporting Information files.*<br>• If neither of these applies but you are able to provide **details of access elsewhere**, with or without limitations, please do so. For example:<br><br>*Data cannot be shared publicly because of [XXX]. Data are available from the XXX Institutional Data Access / Ethics Committee (contact via XXX) for researchers who meet the criteria for access to confidential data.*<br><br>*The data underlying the results presented in the study are available from (include the name of the third party*	All relevant data are within the manuscript and its Supporting Information files.

Powered by Editorial Manager® and ProduXion Manager® from Aries Systems Corporation

and contact information or URL).<br>• This text is appropriate if the data are owned by a third party and authors do not have permission to share the data.<br><br>* typeset	
Additional data availability information:	

Manuscript

Click here to access/download;Manuscript;Manuscript-Ojanguren et al. 2022-Bothriurus mistral.docx

Description of *Bothriurus mistral* n. sp., the highest-dwelling *Bothriurus* from the western Andes (Scorpiones, Bothriuridae), using multiple morphometric approaches

Andrés A. Ojanguren-Affilastro[1,*], Hugo A. Benítez[2,3], Hernán A. Iuri[1], Camilo I. Mattoni[4], Fermín M. Alfaro[5,6,7], Jaime Pizarro-Araya[5,7,8]

1 División Aracnología, Museo Argentino de Ciencias Naturales Bernardino Rivadavia (CONICET), Avenida Ángel Gallardo 470, 1405 DJR, Buenos Aires, Argentina

2 Laboratorio de Ecología y Morfometría Evolutiva, Centro de Investigación de Estudios Avanzados del Maule, Universidad Católica del Maule, Talca 3466706, Chile

3 Instituto Milenio Biodiversidad de Ecosistemas Antárticos y Subantárticos (BASE), Las Palmeras 3425, Santiago, Chile

4 Laboratorio de Biología Reproductiva y Evolución, Instituto de Diversidad y Ecología Animal (IDEA, CONICET–UNC), Facultad de Ciencias Exactas, Físicas y Naturales, Universidad Nacional de Córdoba, Córdoba, Argentina

5 Laboratorio de Entomología Ecológica (LEULS), Departamento de Biología, Facultad de Ciencias, Universidad de La Serena, Casilla 554, La Serena, Chile

6 Instituto de Investigación Multidisciplinar en Ciencia y Tecnología, Universidad de La Serena, Casilla 554, La Serena, Chile

7 Grupo de Artrópodos, Sistema Integrado de Monitoreo y Evaluación de Ecosistemas Forestales Nativos (SIMEF)

8 Instituto de Ecología y Biodiversidad (IEB), Santiago, Chile

* Corresponding author. E-mail addresses: andres.ojanguren@gmail.com & ojanguren@macn.gov.ar

Abstract

We describe *Bothriurus mistral* n. sp. (Scorpiones, Bothriuridae) from the Chilean north-central Andes of the Coquimbo Region. This is the highest elevational discovery for *Bothriurus* in the western slopes of the Andes. This species was collected in the Estero Derecho Private Protected Area and Natural Sanctuary as part of the First National Biodiversity Inventory of Chile of the Integrated System for Monitoring and Evaluation of Native Forest Ecosystems (SIMEF). *Bothriurus mistral* n. sp. is closely related to *Bothriurus coriaceus* Pocock, 1893, from the lowlands of central Chile. This integrative research includes a combination of traditional morphometrics and geometric morphometric analyses to support the taxonomic delimitation of the species.

Introduction

The genus *Bothriurus* is the most widespread taxon among Bothriuridae, occupying most environments of southern South America, from the arid cold areas of southern Patagonia to the tropical rainforests of Amazonia [1]. Despite its high diversity and extended distribution, its presence in high altitudes of the Andes is comparatively marginal [2]. As of now, only three species have been recorded in this environment: *Bothriurus bocki* Kraepelin, 1911 and *Bothriurus trivittatus* Werner, 1939, both from Bolivia and belonging to the *inermis* group [3, 4], and *Bothriurus olaen* Acosta, 1998, from central Argentina and southern Bolivia, belonging to the *burmeisteri* group [5, 6]. All these species occur in the eastern slopes of the Andes, and no species of this genus has ever been collected on the western slopes of this mountain range. This absence is particularly intriguing because several Chilean species of *Bothriurus* occupy arid hilly areas that are environmentally similar to the arid, high Andean slopes [7, 8, 9, 10, 11].

Over the last years our work group has been recording information about the arthropod fauna of Chile via a systematic survey of the whole biota of the country conducted as part of the Integrated System for Monitoring and Evaluation of Native Forest Ecosystems (SIMEF by its Spanish acronym). As a part of this project we have recently performed the first arthropod survey in the protected high-Andean area of the Estero Derecho Private Protected Area and Natural Sanctuary, in the Coquimbo region, central Chile. As a result, our group has managed to collect several species of scorpions, two of which were unknown for the area, including the first high-altitude Andean species of *Bothriurus* ever collected in the western slopes of the Andes.

In this article a combination of methodologies were used to describe *Bothriurus mistral* n. sp. from the Chilean central Andes. This species is most closely related to the

lowland species *Bothriurus coriaceus* Pocock, 1893. Herein we combined traditional taxonomy and geometric morphometrics to provide a better understanding of the morphological trait variation between species, and better support the taxonomic delimitation of the new species.

The presence of this new species in the Andean area of Coquimbo region is remarkable not only because this is the first record of this genus for western Andes, but also because this area has been extensively surveyed in the last two decades [12, 13, 14, 11], with not a single previous record of this genus in the area [11].

Materials and Methods

Study area

The fieldwork was conducted in the Estero Derecho Private Protected Area and Natural Sanctuary, located in the transverse valley range in the northernmost part of the Coquimbo Region, in the Andean area of the Elqui valley (Coquimbo Region, Chile) (Figs 1 and 2B and 2C). The area is located 125 km away from La Serena, upstream from the town of Alcohuaz, and 15 km away from the town of Pisco Elqui [15]. The area is a basin head that provides water from the accumulated winter snow and from multiple rocky glaciers [16]. The large number of high-Andean water meadows present in the area provide water regulation through purification. In addition, the vegetation coverage helps to regulate global-scale climate, temperature and precipitation, as well as erosion, holding the soil in place and preventing landslides. They also harbor a high-Andean biota in good conservation status [15]. In the Andean and high mountainous foothills, the predominant soils are poorly developed entisols and aridisols, which are

more common in the steep slopes of rocky hills with little vegetation coverage [17, 18]. The precipitation in the area is mostly nival, with a yearly average of 200 mm and high interannual variability [18]; between 2,000 masl and 3,000 masl, the climate type is cold mountain steppe, which is characterized by strong winds, high solar radiation, and higher winter precipitation, especially nival.

Fig 1. Distribution map of *Bothriurus mistral* n. sp. (yellow circles) and distribution of *Bothriurus coriaceus* in neighboring areas (red circles). Estero Derecho Private Protected Area and Natural Sanctuary marked with a white line, (Coquimbo Region, Chile).

Fig 2. (A–D) Living specimens and type localities of *Bothriurus mistral* n. sp. herein described. (A) *Bothriurus mistral* n. sp. (B) *Bothriurus mistral* n. sp. under UV light. (C) Type locality of *Bothriurus mistral* n. sp. in the Estero Derecho Private Protected Area and Natural Sanctuary (Coquimbo Region, Chile). (D) Aerial view of the type locality.

Studied material

Studied and measured specimens of *Bothriurus mistral* n. sp. correspond to the type series (see below).

Studied and measured specimens of *Bothriurus coriaceus*: Los Vilos, Coquimbo Province, 30/IX/1983, 2 males, E. Mary coll.; Quebrada Playa Agua Dulce, 45 km N. Los Vilos, Choapa Province, Coquimbo Region, 5–6/XI/1988, 3 males, Maury coll. Same locality and collector, 8/I/1984, 1 male; Cuesta Porotitos, Coquimbo Region,

26/X/2011, 1 male, Ojanguren-Affilastro & Pizarro-Araya Coll.; 30 km N. Ovalle, Coquimbo Region, 10/I/1984, 1 male, Maury coll.; Lampa, Metropolitan Region of Santiago, 19/IX/1977, 2 males, Peña coll.; Guayacán, Metropolitan Region of Santiago, 5/I/1984, 3 males, Maury Coll. Juncal (1,980 masl), Aconcagua Province, Metropolitan Region of Santiago, 5/I/1984, 2 males, Maury coll.

Taxonomic methodology

The descriptive terminology follows Maury [19] for the hemispermatophores; Vachon [20] for the trichobothria; Loria & Prendini [21] for the lateral eyes; Roig-Alsina & Maury [22] for the male telson gland; and Ochoa et al. [23] for the metasomal carinae, which are abbreviated as follows: DL = dorsolateral, LIM = lateral inframedian, LM = lateral median, VL = ventrolateral, VM = ventromedian. The terminology for pedipalp carinae follows Prendini [24] and are abbreviated as follows: DI = dorsal internal, DE = dorsal external, VI = ventral internal, VE = ventral external. The abbreviations for collections are as follows: LEULS: Laboratorio de Entomología Ecológica, Universidad de La Serena, La Serena, Chile (Jaime Pizarro-Araya); MACN: Museo Argentino de Ciencias Naturales “Bernardino Rivadavia”, Buenos Aires, Argentina (Martín Ramírez); MZUC: Museo de Zoología de la Universidad de Concepción, Concepción, Chile (Jorge Artigas); MNHN: Museo Nacional de Historia Natural, Santiago, Chile (Mario Elgueta). All collection sites were georeferenced using a GPS (Garmin® Etrex model). A distribution map (Fig. 1) of *Bothriurus mistral* n. sp. and neighboring records of *B. coriaceus* was generated using ArcMap 9.0 (Enviromental Systems Research Institute [ESRI], Redlands, California); records of *B. coriaceus* were taken from Mattoni & Acosta [10], and more recent collection

records from LEULS. All National and International collection and transport permits were duly obtained, including those provided by the local community managing the Private Protected Area and Natural Sanctuary of Estero Derecho.

Nomenclatorial acts

The electronic edition of this article conforms to the requirements of the amended International Code of Zoological Nomenclature, and hence the new names contained herein are available under that Code from the electronic edition of this article. This published work and the nomenclatural acts it contains have been registered in ZooBank, the online registration system for the ICZN. The ZooBank LSIDs (Life Science Identifiers) can be resolved and the associated information viewed through any standard web browser by appending the LSID to the prefix "http://zoobank.org/". The LSID for this publication is: urn:lsid:zoobank.org:act:1024E801-6EC5-43D8-BC49-F7A5BDD667B1. The electronic edition of this work was published in a journal with an ISSN, and has been archived and is available from the following digital repositories: LOCKSS [author to insert any additional repositories].

Microscopy and images

The measurements, taken using an ocular micrometer, were recorded in millimeters. The hemispermatophores were dissected and then examined in 80% ethanol and photographed under UV and white light. Digital images of pigmentation pattern and habitus were taken under visible light and images of the external morphology under UV light, using a Leica DFC290 digital camera attached to a Leica M165C

stereomicroscope, and the focal planes fused with Helicon Focus 3.10.3 (http://helicon.com.usa/heliconfocus/).

Geometric morphometric approach

In order to have a better understanding of the variation between species, a geometric morphometrics approach was performed using landmarks in the metasomal segment V of the males. We used only males due to a lack of females, and because in this genus it is very difficult to clearly separate adult females from subadults. We chose this morpho-logical trait as a trait with significant taxonomical differences between the species. A total of seve[illegible]n landmarks were digitized (Fig 3), using the software TPSDig2 [25]. The landmarks were aligned using a Procrustes superimposition analysis, by removing size, position and orientation information to standardize the shape of each specimen based on their centroid size [26]. A measurement error (ME) analysis was performed after digitizing the landmarks twice, this data was compared using a Procrustes ANO-VA, in which the mean squares (MS) of error should be smaller than the MS of the in-dividual [27].

Fig 3. General description of the location of the 20 landmarks in the ventral surface of metasomal segment V. (A) *Bothriurus mistral* n. sp. (B) *Bothriurus coriaceus*.

Since the *x* and *y* coordinate were extracted by the Procrustes analysis, the shape variation was analyzed in the entire dataset, using a principal component analysis (PCA) based on the covariance matrix of the individuals’ shape. Since shape changes are asso-

ciated with changes in size, an allometric analysis was performed using a multivariate regression of shape on centroid size [28]. The residuals were used to investigate the shape variation independent of size [29]. To assess whether there are significant differences between the compared species, a random permutation analysis between groups and a Procrustes ANOVA was performed using the RRPP package in R [30].

Results

Taxonomy

Bothriurus mistral n. sp. Ojanguren-Affilastro, Mattoni, Alfaro & Pizarro-Araya
(Figs. 1–9, Table 1)

Type material. Holotype male: Chile, Coquimbo Region, Estero Derecho Private Protected Area and Natural Sanctuary, El Chañar Refuge, 30°23′3.86″S, 70°24′44.97″W, 3,034 masl (SIMEF-Project), 24–26/XI/2020, J. Pizarro-Araya, F.M. Alfaro, J. Calderón, A. Castex (MNHN); Paratypes, Conglomerate no. 41141, 30°22′4.78″S, 70°25′51.30″W, 3,015 masl (SIMEF-Project), 24–26/XI/2020, J. Pizarro-Araya, F.M. Alfaro, J. Calderón, A. Castex, 9 males, 2 females (MACN); 1 male, 6 females, 2 juveniles (MNHN); 1 male, 3 females, 1 juvenile (MZUC); 1 male, 7 females, 2 juveniles (LEULS), Conglomerate no. 41142, 30°21′58.26″S, 70°22′44.63″W, 3,873 masl (SIMEF-Project), 24–26/XI/2020, J. Pizarro-Araya, F.M. Alfaro, J. Calderón, A. Castex, 5 males, 1 female (MACN); 1 male, 4 females, 1

juvenile (MNHN); 2 females, 1 juvenile (MZUC); 1 male, 3 females, 1 juvenile (LEULS).

Etymology. The specific name *mistral* is a noun in apposition referring to Gabriela Mistral, pseudonym of the Chilean poetess Lucila María Godoy Alcayaga (1889–1957), who was born in Vicuña and raised in Monte Grande, both in the Elqui valley (Coquimbo Region), an area adjacent to the type locality of this species. For her poetry work, she was awarded the Nobel Prize in Literature in 1945 and was the first Ibero-American woman and the second Latin American person to receive the Nobel prize. Gabriela Mistral worked as a teacher at numerous schools of the Elqui valley and became a leading thinker as to the role of public education. She was also involved in the reformation of the Mexican education system, and since the 1920s, led an itinerant life due to her work as a consul and representative for international organizations in the Americas and Europe.

Description. Based on the holotype male (MNHN) and paratypes (LEULS, MACN, MNHN, MZUC). Total length, males: 47–57 mm, (N = 10; mean = 52.8 mm); females: 51–55 mm, (N = 3; mean = 52.6 mm). (Measurements of a male and a female paratypes in Table 1).

Tab[illegible]. Measurements (mm) of *Bothriurus mistral* n. sp. (male and female holotype, MNHN).

	Bothriurus mistral n. sp.	
	Male paratype	Female paratype
Total length	50.94	55.93
Carapace, length	6.06	6.22
Carapace, anterior width	3.47	4.36
Carapace, posterior width	6.30	6.63
Mesosoma, total length	15.40	19.56
Metasoma, total length	29.48	30.15
Metasomal segment I, length	3.23	3.64
Metasomal segment I, width	4.44	3.47
Metasomal segment I, height	3.23	2.67
Metasomal segment II, length	3.88	4.20
Metasomal segment II, width	4.20	3.07
Metasomal segment II, height	3.39	2.66
Metasomal segment III, length	4.28	4.44
Metasomal segment III, width	4.04	2.83
Metasomal segment III, height	3.55	2.59
Metasomal segment IV, length	4.85	4.85
Metasomal segment IV, width	4.04	2.75
Metasomal segment IV, height	3.31	2.34
Metasomal segment V, length	6.46	5.99
Metasomal segment V, width	3.88	2.75
Metasomal segment V, height	3.23	2.10
Telson, length	6.78	7.03
Vesicle, length	4.68	3.79
Vesicle, width	2.91	2.42
Vesicle, height	2.02	2.10
Aculeus, length	2.10	3.24
Chela, length	8.88	8.08
Chela, width	3.39	1.77
Chela, height	4.12	2.42

Measurements (mm) of *Bothriurus mistral* n. sp. (male and female paratypes, MNHN).

Colour: Base colour dark yellowish, with dark brown pigmentation pattern in pedipalps, carapace, tergites, metasoma and legs; the remaining yellowish without dark spots (Figs 2A and 4A–4D). Chelicerae with reticular pigmentation on the dorsal margin of manus; external margin of movable finger with a dark spot. Carapace, anterior margin of carapace densely pigmented medially, connecting with two broad dark stripes from the anterior margin to the base of the postocular furrow, lateral margins with an anteromedian wide dark spot, and reticular pigment; posterolateral margins each with a

dark spot, surrounded by reticular pigment; ocular tubercle, and area around the lateral eyes dark brown, almost black. Tergites I–VI each with a single transverse dark stripe that covers most part of the anterior margin of each segment, each with internal unpigmented areas on each side; tergite VII similar to segment VI but with a dark spot in the posterior margin and a dark are on the lateral margins. Sternites III–VI with a thin dark stripe on the lateral margin, the rest unpigmented; sternite VII with a thin dark stripe on the lateral margin, medially with faint pigment pattern. Sternum, genital opercula and pectines unpigmented. Metasomal segments I–III: dorsal surface with a dark area in the posterior margin, and a dark area in the articulation; lateral surfaces pigmented in the posterior half of the segment, between LM and LIM carinae, and connecting with ventral pigment; ventral surface with two thin VL dark stripes and a median wide dark stripe, fusing in the posterior half of the segment, but also connected by reticular pattern in the rest of the segment; segment IV similar to segment III but with dorsal surface without the posterior dark area, and with the pigment pattern of lateral margins reduced to a reticulate pigmentation; segment V: dorsal surface with a dark area in the articulation, and faint pigment in DL surface; lateral margins unpigmented; ventral surface with a thin VM stripe, and two wide VL stripes, which become wider towards the posterior margin, fusing in the posterior third of the segment, but connected to each other by reticulate pigment in the anterior two thirds. Telson, vesicle with faint pigment pattern in the ventral margin, the rest unpigmented, dorsal gland of males, light yellow; aculeus dark brown. Pedipalps: trochanter with faint reticular pigment pattern; femur with well-developed dark stripes along DI, DE, and VE margins, with DI and DE stripes joining in a big dark dorsal spot occupying almost two thirds of the dorsal surface near the articulation with patella. Patella, with dorsointernal and dorsoexternal dark stripes, connected to each other by dense reticulate pattern on

the dorsal surface; internal surface densely pigmented in the dorsal half, ventral surface unpigmented. Chela with faint reticular dark stripes along DI, DM, DS, D, E, V and VM carinae; area near articulation of movable finger, and both fingers, with faint pigment. Legs: coxae and trochanters unpigmented; femora faintly pigmented on the external margin and near articulation with patella; patellae pigmented along external and dorsal margins; tibiae with faint pigment pattern along external and dorsal margins, basitarsi, and telotarsi unpigmented.

Fig. 4. (A–D) *Bothriurus mistral* n. sp., habitus. (A) Male, dorsal aspect, (B) Male, ventral aspect, (C) Female, dorsal aspect, (D) Female, ventral aspect. Scale bars: 1 [illegible].

Morphology. *Chelicerae*: anterior margin of movable fingers strongly curved, more so in males; movable fingers with two small subdistal teeth. *Pedipalps*: Femur with DI, DE carinae granular, extending only half of the segment, VI carinae, granular, extending the entire length of the segment; with one DI, one D, and DE macrosetae; anterior margin with few scattered granules; dorsally with some granules near the anterior margin, the rest of the intercarinal surfaces smooth. Patella, DI and VI carinae formed by an elevation of the tegument, remaining surfaces smooth (Fig 5E); with one DI and one VI macrosetae. Chela manus (Figs 5A–5D and 5F) robust, length/height ratio, males: 2.02–2.34 (N = 10; mean = 2.23), females: 2.59–2.60 (N = 3; mean = 2.59); length/width, males: 2.47–2.78 (N = 10; mean = 2.64); females: 2.97–3.16 (N = 3; mean = 3.06); internal surface smooth in females (Fig 5F), or with a pronounced, subtriangular projection near articulation of movable finger in males (Figs 5A and 5B and 5D); fingers comparatively short, with a median row of denticles; fixed fingers with six pairs of accessory denticles; movable finger with five external accessory denticles and six

internal accessory denticles; the basal external denticle is usually part of the median row. Trichobothrial pattern neobothriotaxic major type C, with one accessory trichobothrium in *V* series of chela; femur with 3 trichobothria (*d*, *i*, *e*), one macroseta (M1) between *d* and *i*; patella with 19 trichobothria (2 *d*, *i*, 3 *et*, *est*, 2 *em*, 2 *esb*, 5 *eb*, 3 *V*), with *esb*2 petite; chela with 27 trichobothria (*Dt*, *Db*, 5 *Et*, *Est*, *Esb*, 3 *Eb*, *dt*, *dst*, *dsb*, *db*, *et*, *est*, *esb*, *eb*, *ib*, *it*, 5 *V*), with *Et4* petite, *Esb* forming triangle with *Eb*1 and *Eb*2. *Carapace*: anterior margin slightly convex, with two median setae. Surface: finely granular in the dorsolateral margins in males, smooth in females. Anterior longitudinal sulcus absent, postocular furrow present and well developed, lateral sulci shallow. Median ocular tubercle well developed, placed in the middle of the carapace; interocular sulcus barely visible; median ocelli well developed, facing towards the lateral margins, *ca*. two diameters apart, with one seta behind each eye. Lateral ocelli pattern type 3A; with three small lateral ocelli on each side of carapace, two of them anteriorly, third ocellus similar in size to the other two, about one diameter above them. *Legs*: Surfaces smooth. Basitarsi each with two well developed symmetrical pedal spurs. Telotarsi, ventrally with a ventromedian row hyaline setae, and paired ventrosubmedian spines: 1/1 in telotarsus I, 2/2 in telotarsus II and 3/3 in telotarsi III and IV. Ungues curved and short.

Fig 5. (A–F) *Bothriurus mistral* n. sp. (A) Left pedipalp chela, male, internal aspect, (B) Left pedipalp chela, male, ventral aspect, (C) Left pedipalp chela, male, external aspect, (D) Left pedipalp chela, male, dorsal aspect, (E) left pedipalp femur, male, external aspect, (F) Left pedipalp chela, female, ventrointernal aspect. Scale bars: 1 mm.

Pectines: Well developed. Tooth count, males: 15–18 (N = 10; median = 17); females: 13–15 (N = 3; median = 14). *Sternum*: With two conspicuous subtriangular lateral lobes clearly connecting medially, with two macrosetae on each. *Genital opercula*: Sclerites subtriangular. *Tergites*: I–VI: surface smooth; tergite VII with posterior margin finely granular, and four carinae reduced to a single posterior granule each. *Sternites*: III–VI surface finely granular medially in males, smooth in females, with small elliptical spiracles; sternite VII finely granular medially in males, more densely granular in females, with four poorly developed VSM carinae in females. *Metasoma*: segment I dorsal surface smooth, DL carina formed by scattered granules in the posterior two thirds of the segment, with one DL setae; lateral surface: LSM and LIM carinae restricted to the posterior half of the segment, with some scattered granules between DL and LSM carinae, with one LSM and one LIM setae; ventral surface: females, with four longitudinal carinae occupying the entire length of the segment, two VL and two VSM (Fig 6G); males with smooth tegument (Fig 6H); with eight ventral macrosetae arranged in an anterior row of four macrosetae (two VSM and two VL) and a similar row of four setae in the posterior margin of the segment; segment II similar to segment I, but with a DL macroseta, without LIM macroseta, less granular and with less developed carinae, ventral surface in males and females smooth; segment III: as segment II but even less granular and with less developed carinae, DL and LSM carina restricted to some blunt granules in the posterior margin of the segment, LIM carina absent, reduced to a LIM macroseta, ventral surface smooth, the rest as in segment II; segment IV: surfaces smooth, except for some DL granules in males, macrosetae as segment III; segment V: elongated, length/width, males: 1.80–2.01 (N = 10; mean = 1.86); females: 1.73–1.79 (N = 3; mean = 1.75); dorsal surface smooth, medially with a conspicuous furrow, lateral surfaces smooth, with one DL and three LSM macrosetae, ventral surface with

granular tegument (Figs 6C and E), VL carinae granular, extending the entire length of the segment, with three VL macrosetae (Fig 6A), VSM carinae longitudinal in the anterior half of the segment, diverging in the posterior half of the segment and connecting with the VL carinae in the posterior quarter, with three rows of two VSM macrosetae each, VM carina longitudinal, occupying almost the entire length of the segment; anal arch with four macrosetae. *Telson*: vesicle globose (Figs 6A and 6b), being more elongated in males (Fig 6B), ventral surface granular, dorsal surface smooth, in males there is a median conspicuous dorsal tegumentary depression that corresponds to the telson gland; aculeus short and curved, more elongated in males, base of the aculeus comparatively wide and with a small inconspicuous ventral granule.

Fig. 6. (A–J) (A–C, E, G, H). *Bothriurus mistral* n. sp. (A) Telson and metasomal segment V, female, lateral aspect; (B) Telson, male, lateral aspect; (C) Metasomal segment V, female, ventral aspect; (E) Metasomal segment V, male, ventral aspect; (G) Sternite VII and metasomal segments I and II, female, ventral aspect; (H) Sternite VII and metasomal segments I and II, male, ventral aspect; **(D, F–J) *Bothriurus coriaceus*.** (D) Telson, male, lateral aspect; (F) Metasomal segment V, male, ventral aspect; (I) Sternite VII and metasomal segments I and II, female, ventral aspect; (J) Sternite VII and metasomal segments I and II, male, ventral aspect. Scale bars: 1 mm.

Hemispermatophore: distal lamina slender, similar in size to the basal portion, slightly curved in its distal third (Figs 7B and 7C); distal crest parallel to the posterior margin, divided by a transversal ridge and occupying less than the distal quarter of the distal lamina; internal lobe with an external apophysis (Fig 7D), we did not observe any small

granule nor crest in the depression between the apophysis and the frontal ridge; basal lobe laminar, distally bilobular; frontal ridge well developed; capsular cavity well developed (Fig 7E).

Fig. 7. Hemispermatophore of *Bothriurus coriaceus* and *Bothriurus mistral* n. sp. (A–E) (A) *Bothriurus coriaceus*, left hemispermatophore, external aspect. (B–E) *Bothriurus mistral* n. sp. Left hemispermatophore (B) External aspect. (C) Internal aspect. (D) Detail of the internal lobe. (E) Detail of lobe region. Scale bars: 1 mm.

Diagnosis and comparisons. *Bothriurus mistral* n. sp. is most closely related to *B. coriaceus*, which occurs in neighboring areas but at much lower altitudes. Both species can be easily told apart by their pigment pattern and morphology. *Bothriurus mistral* n. sp. is more pigmented than *B. coriaceus*; the pigment pattern of tergites I–VI is usually restricted to the anterior third of the segment in *B. coriaceus*, whereas in *B. mistral* n. sp. it occupies more than the anterior half of each segment (Fig 4A and 4C). Additionally, *B. coriaceus* only has a median wide stripe in the ventral surface of metasomal segments I–IV, whereas *B. mistral* n. sp. has three ventral stripes, two VL and a VM (Fig 4D). *Bothriurus coriaceus* has more developed ventral carinae in metasomal segment I (Fig 6G–6J). Metasomal segment V is more elongated in *B. mistral* n. sp. males (Fig 6E), and its length/width ratio ranges from 1.80 to 2.01 (N = 10; mean = 1.86), whereas in *B. coriaceus* males (Fig 6F) it ranges from 1.61 to 1.76 (N = 10; mean = 1.67). The base of the aculeus in *B. mistral* n. sp. males is slightly thicker and higher than in *B. coriaceus* males (Figs 6B and 6D), but this character is highly variable. There are also some conspicuous differences in the hemispermatophores of both species; in *B. mistral* n. sp. the distal lamina is more

elongated and more recurved apically than in *B. coriaceus* (Figs 7A–7B), being the distal lamina proportionally shorter and wider in *B. coriaceus* than in *B. mistral* n. sp. considering the total length of the hemispermatophore.

Distribution. *Bothriurus mistral* n. sp. is only known from its type locality, the Estero Derecho Private Protected Area and Natural Sanctuary (30°23′3.86″S, 70°24′44.97″O, 3,034 masl), located in the Paihuano mountain range, an area with a surface of 31,680 ha, placed at high altitudes of the north central Chilean Andes (Coquimbo Region, Chile) (Fig 1).

Ecology. The area where *Bothriurus mistral* n. sp. was collected occupies the sub-Andean floor, which is characterized by the presence of medium-height shrub vegetation and scrublands, with the dominant species *Stipa chrysophylla*, *Viviana marifolia*, *Cristaria andicola*, *Adesmia hystrix* Philippi, 1860 (Fabaceae), and *Ephedra americana* Humb. & Bonpl. ex Willd.; and the lowermost Andean floor, characterized by a low tropical-Mediterranean shrubland of *Adesmia subterranea* Clos, Gay, C., 1838, and *Adesmia echinus* C. Presl, 1791 (Fabaceae) [31, 15].

Bothriurus mistral n. sp. has been found in sympatry with *Brachistosternus gayi* Ojanguren-Affilastro, Pizarro-Araya & Ochoa, 2018, and with an undetermined, possibly new, species of *Brachistosternus*.

Geometric morphometric analyses

The measurement error was assessed to avoid any digitizing error in the data. The results of a Procrustes ANOVA showed that the MS values of the individuals exceeded

the MS values of error, indicating that there is no ME in the data. The PCA between species showed that the first three components explained 85.9% of the variation in shape between *B. coriaceus* and *B. mistral* n. sp. (PC1: 68.1%; PC2: 12.4%; PC3: 5.43%). These results showed that almost all the variation was concentrated in the first dimension of the shape space and could be a size effect. Therefore, a multivariate regression between shape and centroid size was performed that showed a significant size effect of 25% ($P < 0.001$, after 10,000 permutation rounds) (Fig 8).

Fig 8. Multivariate regression of centroid size (independent variable) and shape (dependent variable) between *B. coriaceus* and *B. mistral* n. sp. The colors represent the different species (blue and orange respectively).

After removing all size (allometric) effect, the PCA of the residuals showed a new shape variation explained by the first three components with a 82% (PC1: 59.6%; PC2: 16.1%; PC3: 6.4%) that suggests that the shape variation between *B. coriaceus* and *B. mistral* n. sp. was purely a shape variation (Fig 9).

Fig 9. Principal component analysis of the multivariate regression residuals of the metasomal segment V shape between *B. mistral* n. sp. and *B. coriaceus,* including the average shape wireframe between the species. The colors of the confidence ellipses represent: orange: *B. mistral* n. sp., blue: *B. coriaceus.*

Discussion

Bothriurus mistral n. sp. belongs to the *coriaceus* group, which is defined in this contribution and also includes *B. coriaceus* from north-central Chile, *Bothriurus keyserlingi* Pocock, 1893 from south-central Chile, and *Bothriurus dumayi* Cekalovic, 1974 from northern Chile [11]. These species occur in lowlands, or at most, at intermediate altitudes, but never above 2,000 masl. [10]. *Bothriurus mistral* n. sp. is the first known high-altitude *Bothriurus* species from the western slopes of the Andes and the first high-altitude species of the *coriaceus* group. This species seems to be very restricted geographically, being only known from its type locality, despite occurring in a relatively well studied area of the Chilean central Andes [12, 13, 14]. The lack of previous records of this species could be due to the particular characteristics of the environment where it has been collected, which is an inter-Andean valley surrounding a wetland, a kind of environment usually poorly preserved due to herding, and therefore neglected in arthropod surveys. In this case the exclusion of livestock in the area has preserved an almost pristine environment, representing a rare opportunity to study this kind of Andean wetland. Other neighboring areas with similar environments to the type locality should be surveyed in the future in order to clearly establish the actual distribution of this species. This is a further confirmation that the arthropod fauna of high-altitude environments of the Andes is far more complex and diverse than previously thought.

For this research the inclusion of geometric morphometrics analysis of the meta-soma results to be very informative and powerful to discriminate cryptic morphologies [32]. Geometric morphometrics tools have been rarely used in scorpions. Traditionally, some authors used it to evaluate Ecomorphs (morphologies under similar ecological demands) [33, 34], postembryonic stages [35] or in a paleontological way [36], being

this contribution the first to use these techniques to improve a taxonomical description in the group.

This is the first time that a geometric morphometric approach is applied to identify two closely related species of Bothriuridae. Our analyses showed very promising results and these techniques could be implemented for other species of the group in the future, specially to solve the systematic position of taxonomically problematic species.

Acknowledgements

We are grateful to the community of Estero Derecho Private Protected Area and Natural Sanctuary, and to esteroderecho.cl (Coquimbo Region, Chile) for providing field support and logistics access to their plots. We are also grateful to Juan Calderón (ULS) and Alberto Castex (fundacion-indomita.org) for providing field logistics support, and to Francisco A. Squeo and Lutgarda "Lutty" Arriagada (ULS-IEB) for helping to manage the field work in Estero Derecho Private Protected Area and Natural Sanctuary. We also thank Gerardo Vergara (INFOR) for managing the financing of the project Integrated Forest Ecosystem Assessment and Monitoring System (SIMEF by its Spanish acronym). AAOA thanks the PICT 2019-597 project by Agencia Nacional de Promoción Científica y Tecnológica (Argentina). JPA thanks the DIDULS PR2121210 and DIDULS PEQMEN212124 projects of the University of La Serena, Chile, and the funding from the Ministry of Education of Chile, through MINEDUC's performance agreement: Implementation of a competitive model of innovation and creation: preparing the University of La Serena for 2030, ULS19101, ANID FB210006 grant, and the SIMEF project (INFOR-IEB Agreement).

References

1. Mattoni CI. The genus *Bothriurus* (Scorpiones, Bothriuridae) from Patagonia. Insect Syst & Evol. 2007; 38:173–192.
2. Ojanguren-Affilastro AA, Kovarík F. Bothriuridae. In: Kovarík F, Ojanguren-Affilastro AA. Illustrated catalog of scorpions. Part II. Bothriuridae; Chaerilidae; Buthidae I., genera *Compsobuthus*, *Hottentotta*, *Isometrus*, *Lychas*, and *Sassanidotus*. Jakub Rolčík Publisher, Czech Republic, 2013. p. 6-130.
3. Maury EA. A new *Bothriurus* from Bolivia (Scorpiones, Bothriuridae). Bull Am Mus Nat Hist 1981; 170(1):29–33.
4. Maury EA. Redescripción de *Bothriurus bocki* Kraepelin 1911 (Scorpiones, Bothriuridae). J Arachnol. 1984; 12(3):351–356.
5. Acosta LE. Descripción de *Bothriurus olaen*, nueva especie de escorpión de Argentina central (Scorpiones, Bothriuridae). Rev Arachnol. 1997; 12(1):1–8.
6. Acosta LE, Ochoa JA. Lista de los escorpiones bolivianos (Chelicerata: Scorpiones), con notas sobre su distribución. Rev Soc Entomol Argent. 2002; 61(3–4):15–23.
7. Mattoni CI. *Bothriurus picunche* sp. nov., a new scorpion from Chile (Scorpiones, Bothriuridae). Stud Neotrop Fauna Environ. 2002a; 37(2):169–174.
8. Mattoni CI. La verdadera identidad de *Bothriurus vittatus* (Guérin-Méneville, [1838]) (Scorpiones, Bothriuridae). Rev. Arachnol. 2002b; 14(5):59–72.
9. Mattoni CI. *Bothriurus pichicuy* sp. nov., nuevo escorpión chileno del grupo *vittatus* (Scorpiones: Bothriuridae). Iheringia Ser. Zool. 2002c; 92(4):81–87.

10. Mattoni CI, Acosta LE. Systematics and distribution of three *Bothriurus* species (Scorpiones, Bothriuridae) from central and northern Chile. Stud Neotrop Fauna Environ. 2006; 41 (3):235–250.

11. Pizarro-Araya J, Ojanguren-Affilastro AA. El orden Scorpiones en Chile: estado del conocimiento, desde la taxonomía a la conservación. Revista Parasitología Latinoamericana, Numero especial Arácnidos comunes de Chile 2021; II: 23–49.

12. Ochoa JA, Ojanguren-Affilastro AA, Mattoni CI, Prendini L. Systematic revision of the Andean scorpion genus *Orobothriurus* Maury, 1976 (Bothriuridae), with discussion of the altitude record for scorpions. Bull Am Mus Nat Hist. 2011; 359:1-90.

13. Ojanguren-Affilastro AA, Mattoni CI. A new species of *Brachistosternus* from Chilean central Andes (Scorpiones: Bothriuridae). Stud Neotrop Fauna Environ. 2006; 41:79–85.

14. Ojanguren-Affilastro AA, Agusto P, Pizarro-Araya J, Mattoni CI. Two new scorpion species of genus *Brachistosternus* (Scorpiones: Bothriuridae) from northern Chile. Zootaxa. 2007; 1623:55–68. https://doi.org/10.11646/zootaxa.1623.1.4

15. Comunidad Agrícola Estancia Estero Derecho. Plan de Manejo para la Conservación del Área Protegida Privada y Santuario de la Naturaleza Estero Derecho, Pisco Elqui, Región de Coquimbo, Chile. 2017. pp 77.

16. de Pasquale G, Valois R, Schaffer N, MacDonell S. Contrasting geophysical signatures of a relict and an intact Andean rock glacier. Cryosphere. 2022; 16:1579–1596. https://doi.org/10.5194/tc-16-1579-2022

17. Squeo FA, Veit H, Arancio G, Gutiérrez JR, Arroyo MTK, Olivares N. Spatial heterogeneity of high mountain vegetation in the andean desert zone of Chile (30°S). Mt Res Dev. 1993; 13:203–209. https://doi.org/10.2307/3673638

18. Cepeda J, Novoa J. La cordillera altoandina del valle de Elqui. In: Cepeda J, editor. Geoecología de los Andes desérticos: la alta montaña del valle del Elqui. Ediciones Universidad de La Serena, La Serena, Chile. 2006. p. 41–63.

19. Maury EA. La estructura del espermatóforo en el género *Brachistosternus* (Scorpiones, Bothriuridae). Physis (Bs As) sec. C. 1975; 34(89):179–182.

20. Vachon M. Étude des caractères utilisés pour classer les familles et les genres de scorpions (Arachnides). 1. La trichobothriotaxie en arachnologie. Sigles trichobothriaux et types de trichobothriotaxie chez les scorpions. Bull Mus Hist Nat Paris. 3e sér. 1973. [1974]; 140:857–958.

21. Loria S, Prendini L. Homology of the lateral eyes of Scorpiones: A six-ocellus model. PLoS One. 2014; 9(12):e112913. https://doi.org/10.1371/journal.pone.0112913

22. Roig-Alsina AH, Maury EA. Consideraciones sistemáticas y ecológicas sobre *Brachistosternus* (*Leptosternus*) *borellii* Kraepelin, 1911 (Scorpiones, Bothriuridae). Physis, Sección C. 1981; 39:1–9.

23. Ochoa JA, Botero-Trujillo R, Prendini L. On the troglomorphic scorpion *Troglotayosicus humiculum* (Scorpiones, Troglotayosicidae), with first description of the adults. Am Mus Nov. 2010; 3691:1‑19. https://doi.org/10.1206/707.1

24. Prendini L. Phylogeny and classification of the superfamily Scorpionoidea Latreille, 1802 (Chelicerata, Scorpiones): an exemplar approach. Cladistics 2000; 16:1–78. https://doi.org/10.1006/clad.1999.0127

25. Rohlf FJ. TpsDig, version 2.17. Department of Ecology and Evolution, State University of New York at Stony Brook, Nueva York, 2013.

26. Rohlf FJ, Slice D. Extensions of the Procustes methods for the optimal superimposition of landmarks. Syst Zool 1990; 39:40-59. https://doi.org/10.2307/2992207

27. Fruciano C. Measurment error in geometric morphometrics. Dev Genes Evol. 2016; 226:139–158. https://doi.org/10.1007/s00427-016-0537-4

28. Monteiro LR. Multivariate regression models and geometric morphometrics: The search for causal factors in the analysis of shape. Syst Biol. 1999; 48:192–199. https://doi.org/10.1080/106351599260526

29. Benítez HA, Bravi R, Parra LE, Sanzana M-J, Sepúlveda-Zúñiga E. Allometric and non-allometric patterns in sexual dimorphism discrimination of wing shape in *Ophion intricatus*: Might two male morphotypes coexist? J Insect Sci. 2013; 13:143. https://doi.org/10.1673/031.013.14301

30. Collyer ML, Adams DC. RRPP: An r package for fitting linear models to high-dimensional data using residual randomization. Methods Ecol Evol. 2018; 9:1772–1779. https://doi.org/10.1111/2041-210X.13029

31. Squeo FA, Osorio R, Arancio G. Flora de los Andes de Coquimbo: Cordillera de Doña Ana. Universidad de La Serena, La Serena, Chile. 1994; pp. 176.

32. Benítez HA, Pizarro-Araya J, Bravi R, Sanzana M-J, Alfaro FM. Morphological variation on isolated populations of *Praocis* (*Praocis*) *spinolai*. J Insect Sci. 2014; 14:1–12. https://doi.org/10.1093/jis/14.1.11

33. Coelho P, Kaliontzopoulou A, Rasko M, van der Meijden A. A ‘striking’ relationship: scorpion defensive behaviour and its relation to morphology and performance. Funct Ecol. 2017; 31:1390–1404. https://doi.org/10.1111/1365-2435.12855

34. Coelho P, Kaliontzopoulou A, Sousa P, Stockmann M, van der Meijden A. Reevaluating scorpion ecomorphs using a naïve approach. BMC Ecol Evol. 2022; 22:1–10. https://doi.org/10.1186/s12862-022-01968-0

35. Gómez M, Flórez E, Hernández L, Medina GEF. Etapas de desarrollo post embrionario de *Tityus columbianus* (Thorell, 1846) (Scorpiones, Buthidae): enfoque de estimación morfométrica. Mutis. 2017; 7:75.

36. Bicknell RDC, Amati L. On the morphospace of *eurypterine* sea scorpions. Earth Environ Sci Trans R Soc Edinb. 2022; 113:1–6. https://doi.org/10.1017/S175569102100030X

Figure 1

Click here to access/download;Figure;Fig 1-Ojanguren et al. 2022-Bothriurus mistral.tif

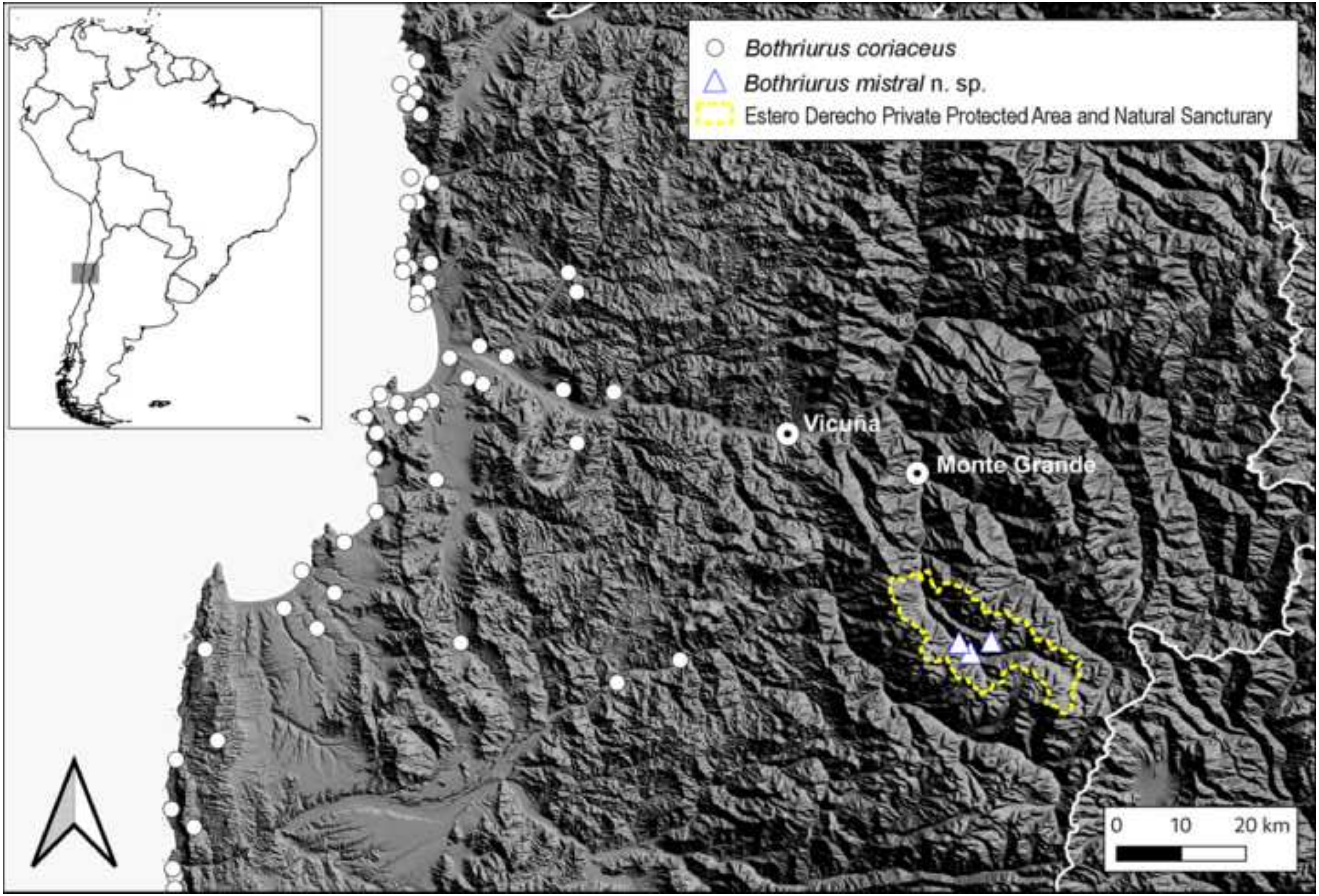

Figure 2

Click here to access/download;Figure;Fig 2-Ojanguren et al. 2022-Bothriurus mistral.tif

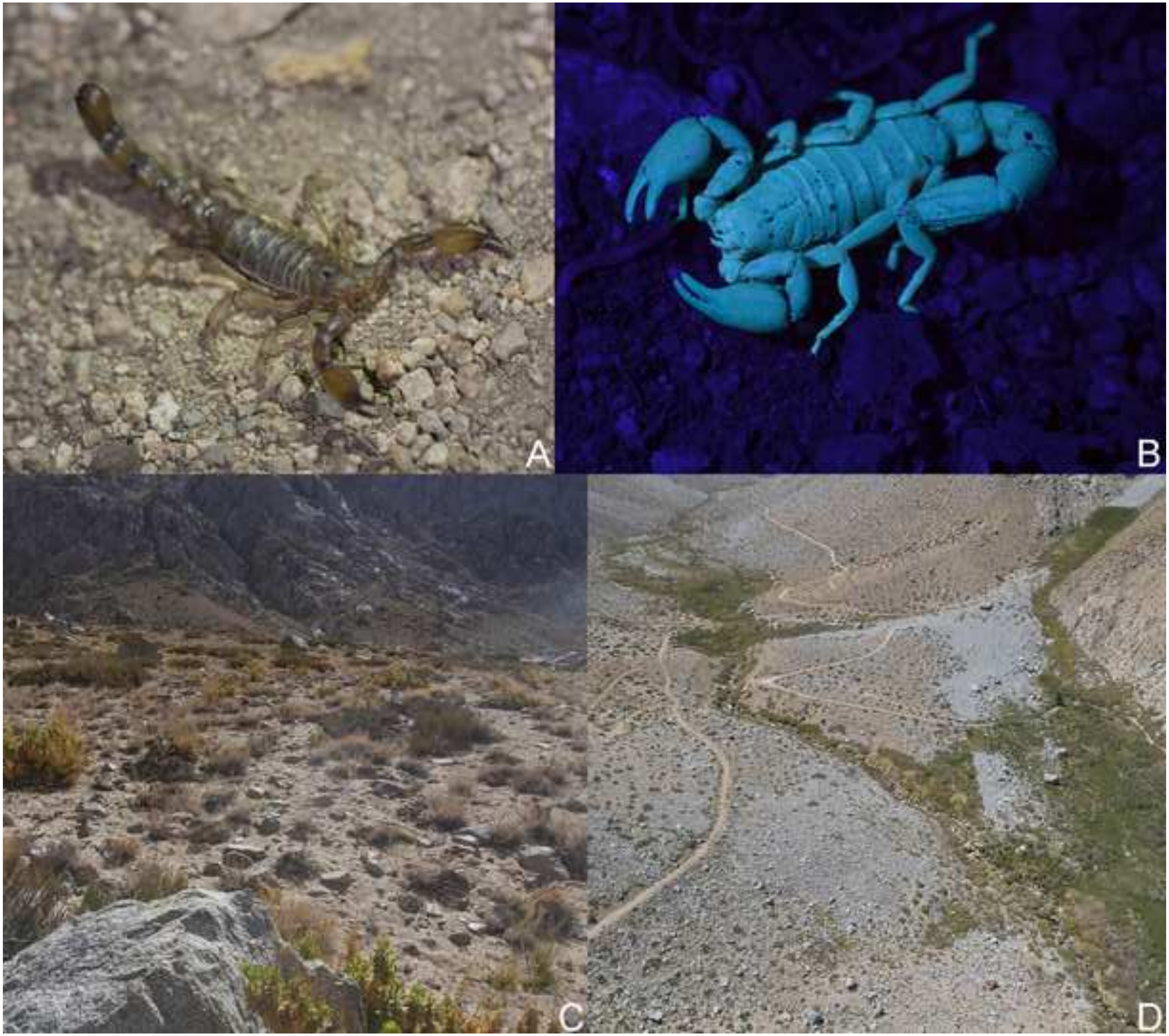

Figure 3

Click here to access/download;Figure;Fig 3-Ojanguren et al. 2022-Bothriurus mistral.tif

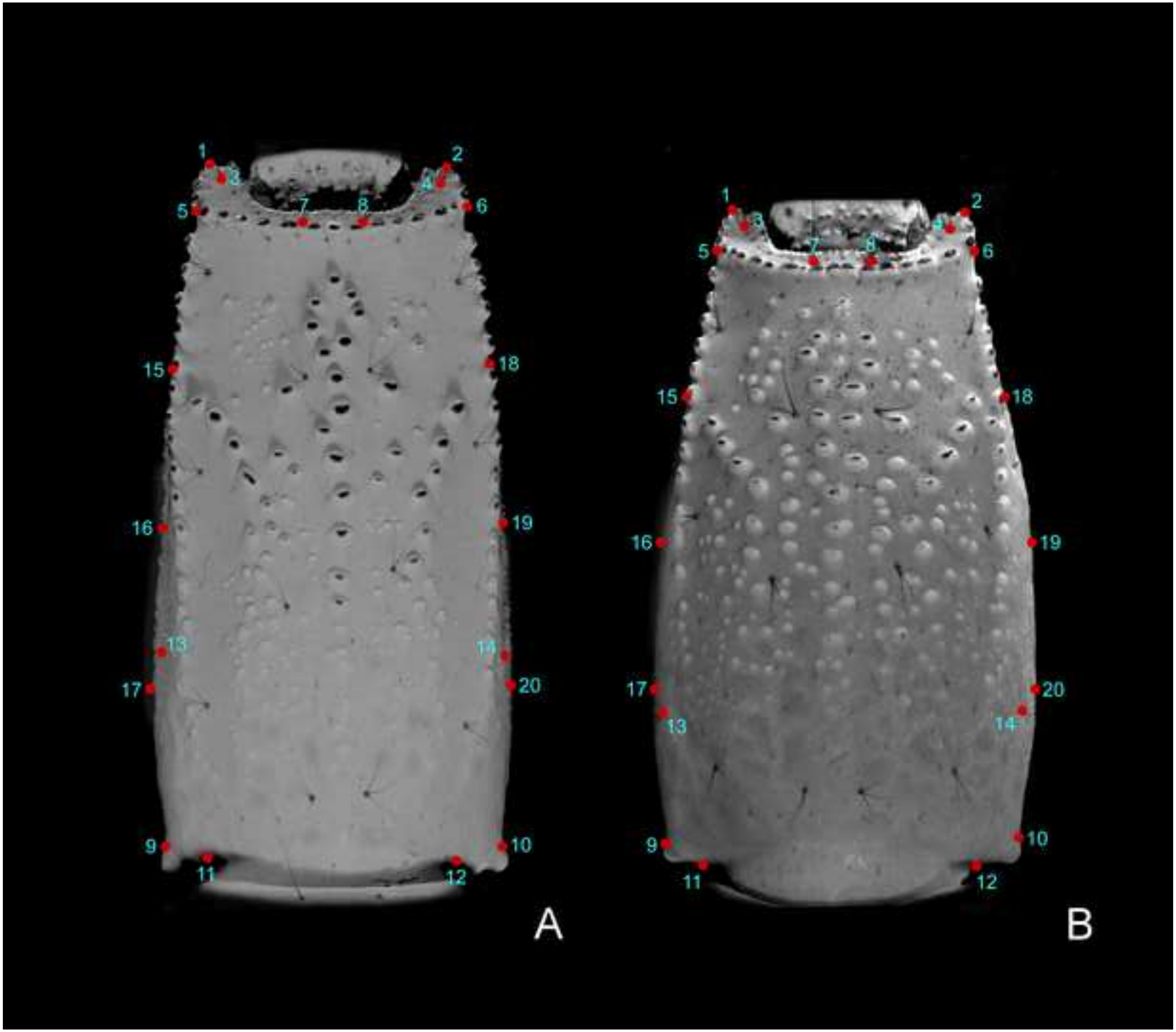

Figure 4

Click here to access/download;Figure;Fig 4-Ojanguren et al. 2022-Bothriurus mistral.tif

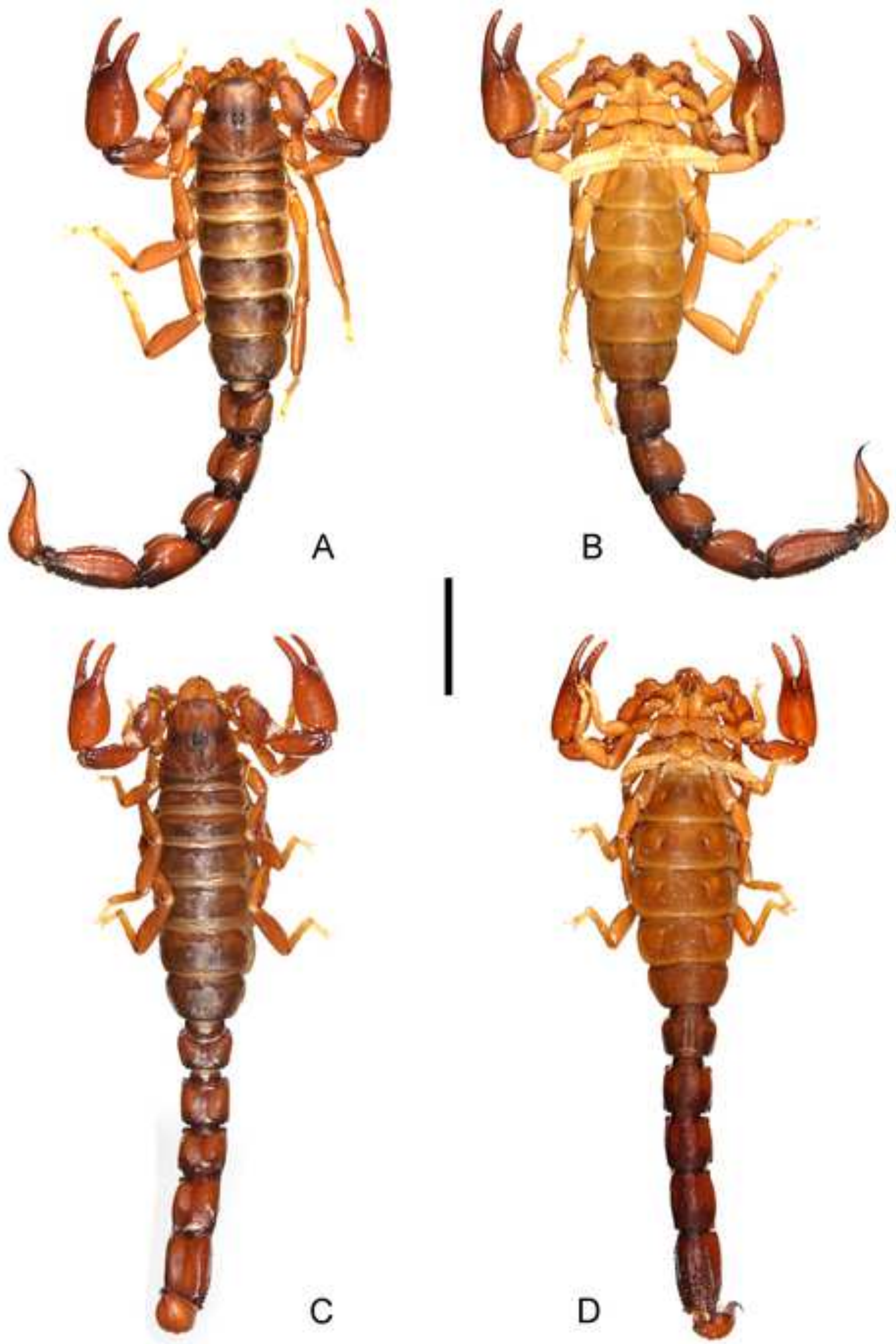

Figure 5

Click here to access/download;Figure;Fig 5-Ojanguren et al. 2022-Bothriurus mistral.tif

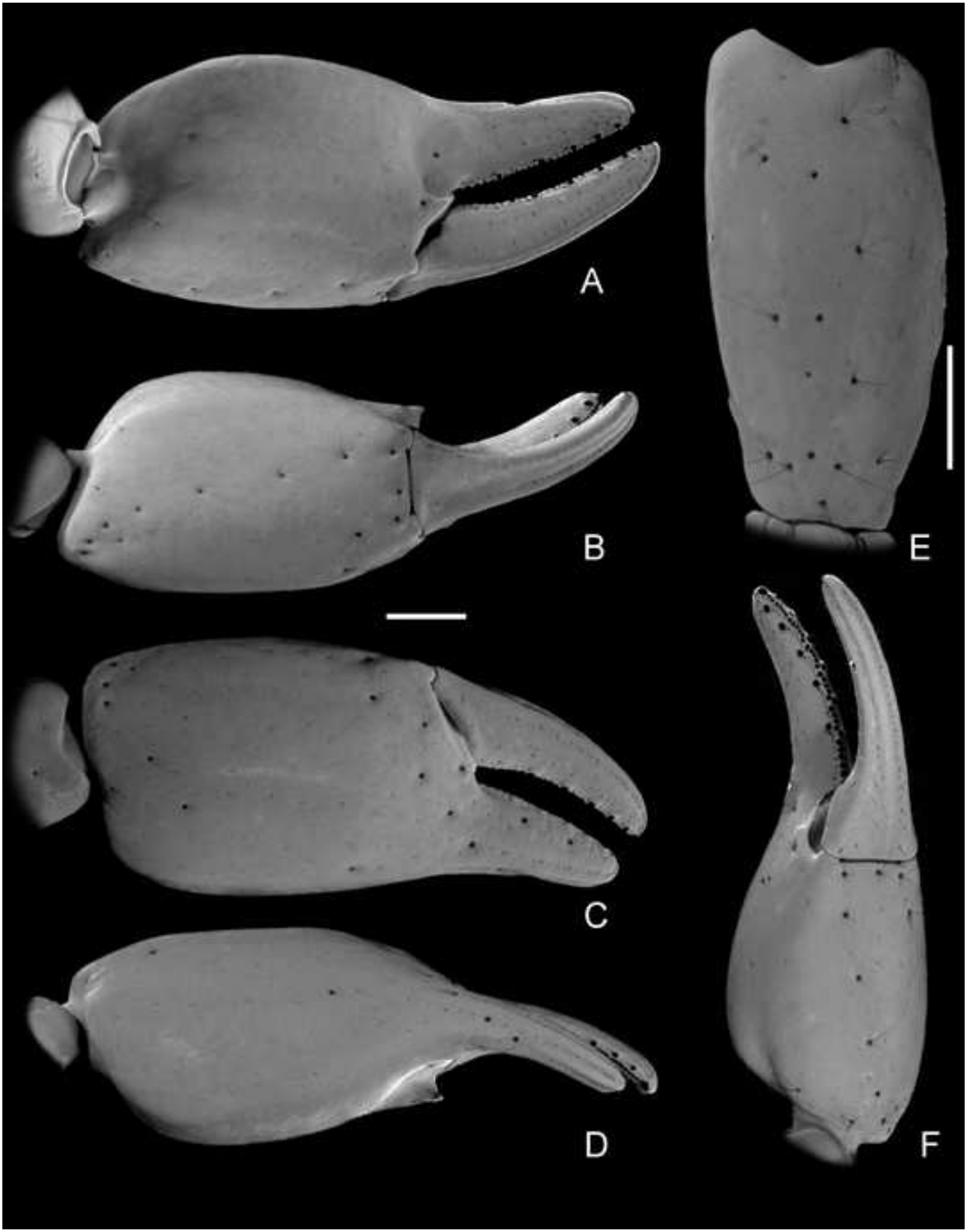

Figure 6

Click here to access/download;Figure;Fig 6-Ojanguren et al. 2022-Bothriurus mistral.tif

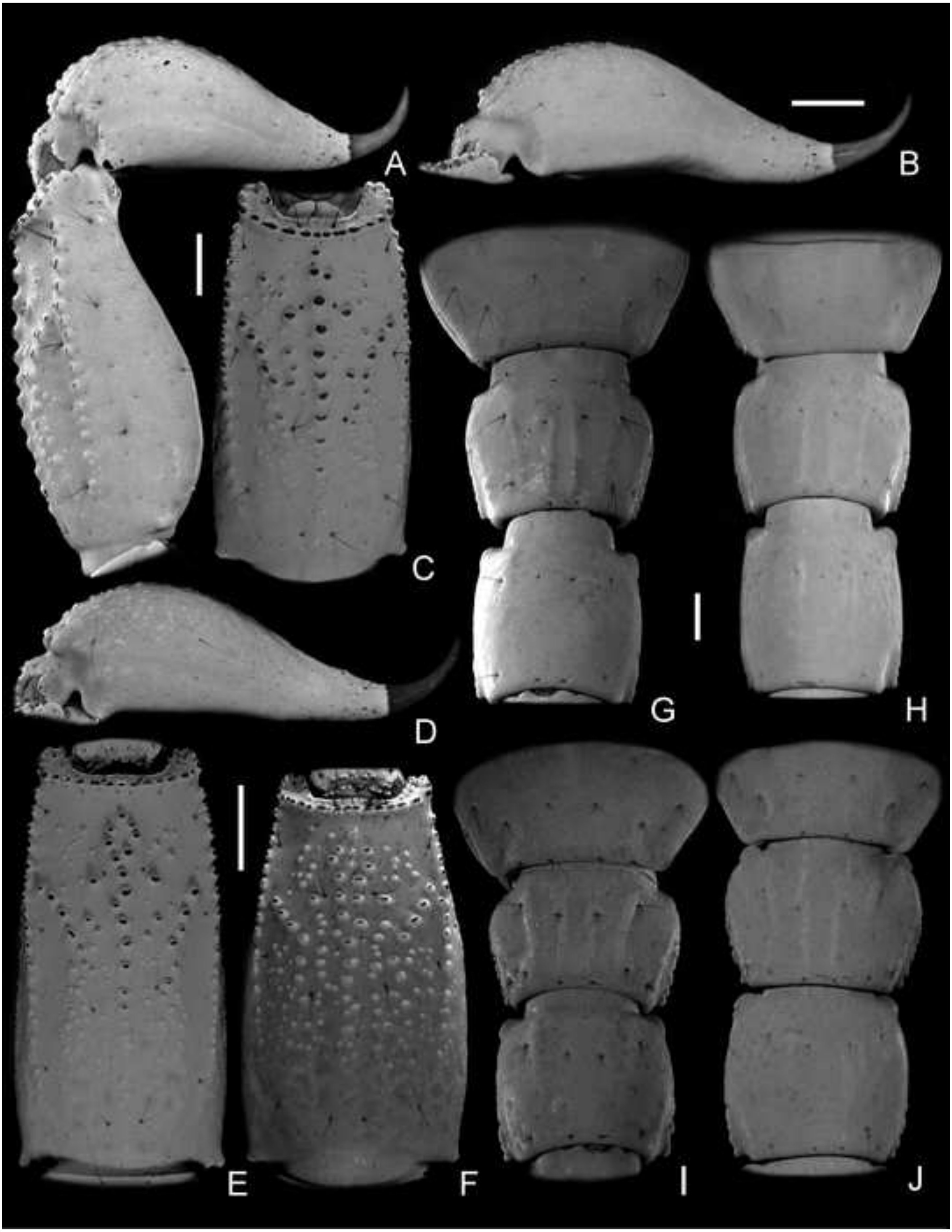

Figure 7

Click here to access/download;Figure;Fig 7-Ojanguren et al. 2022-Bothriurus mistral.tif

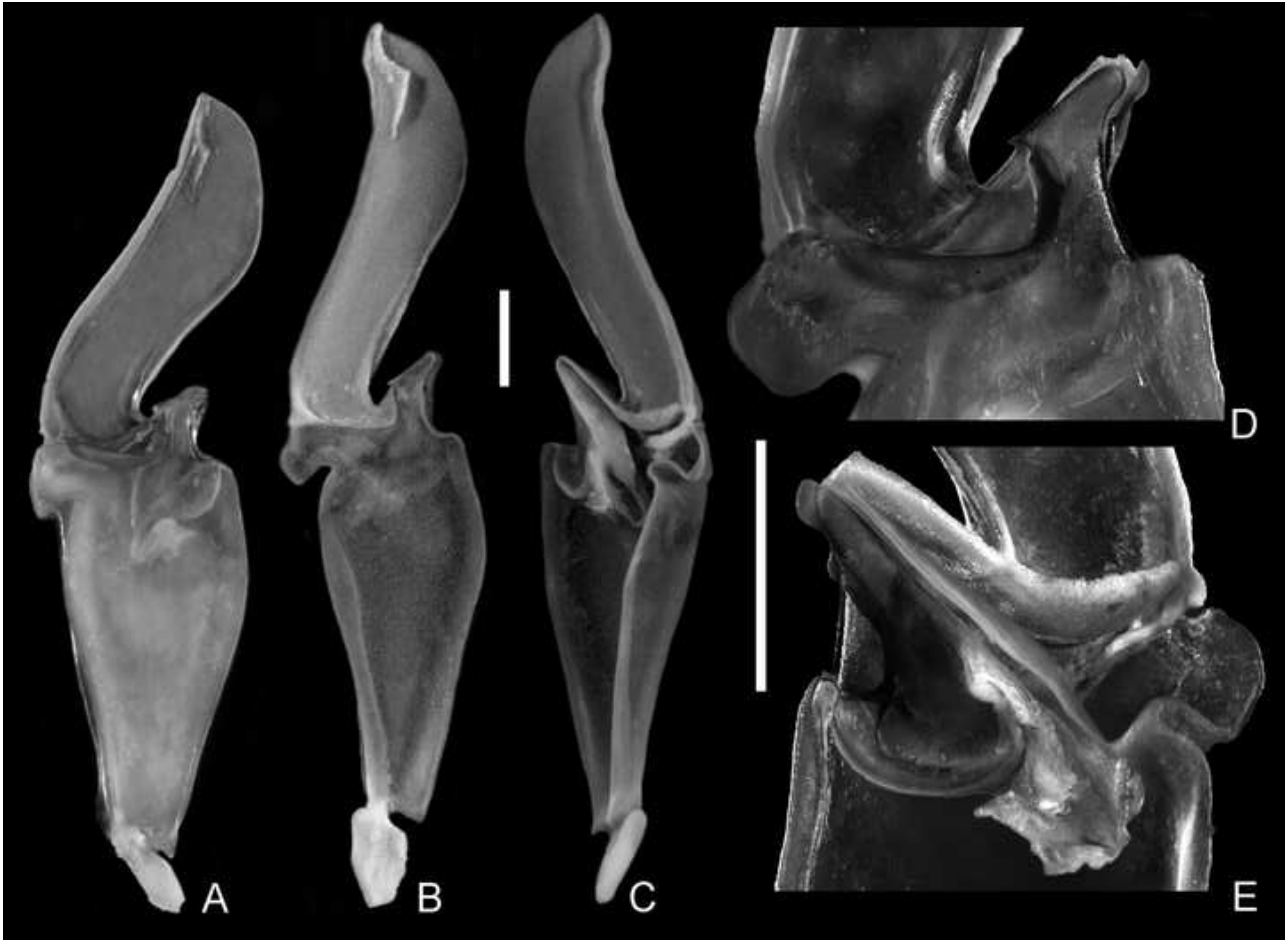

Figure 8

Click here to access/download;Figure;Fig 8-Ojanguren et al. 2022-Bothriurus mistral.tif

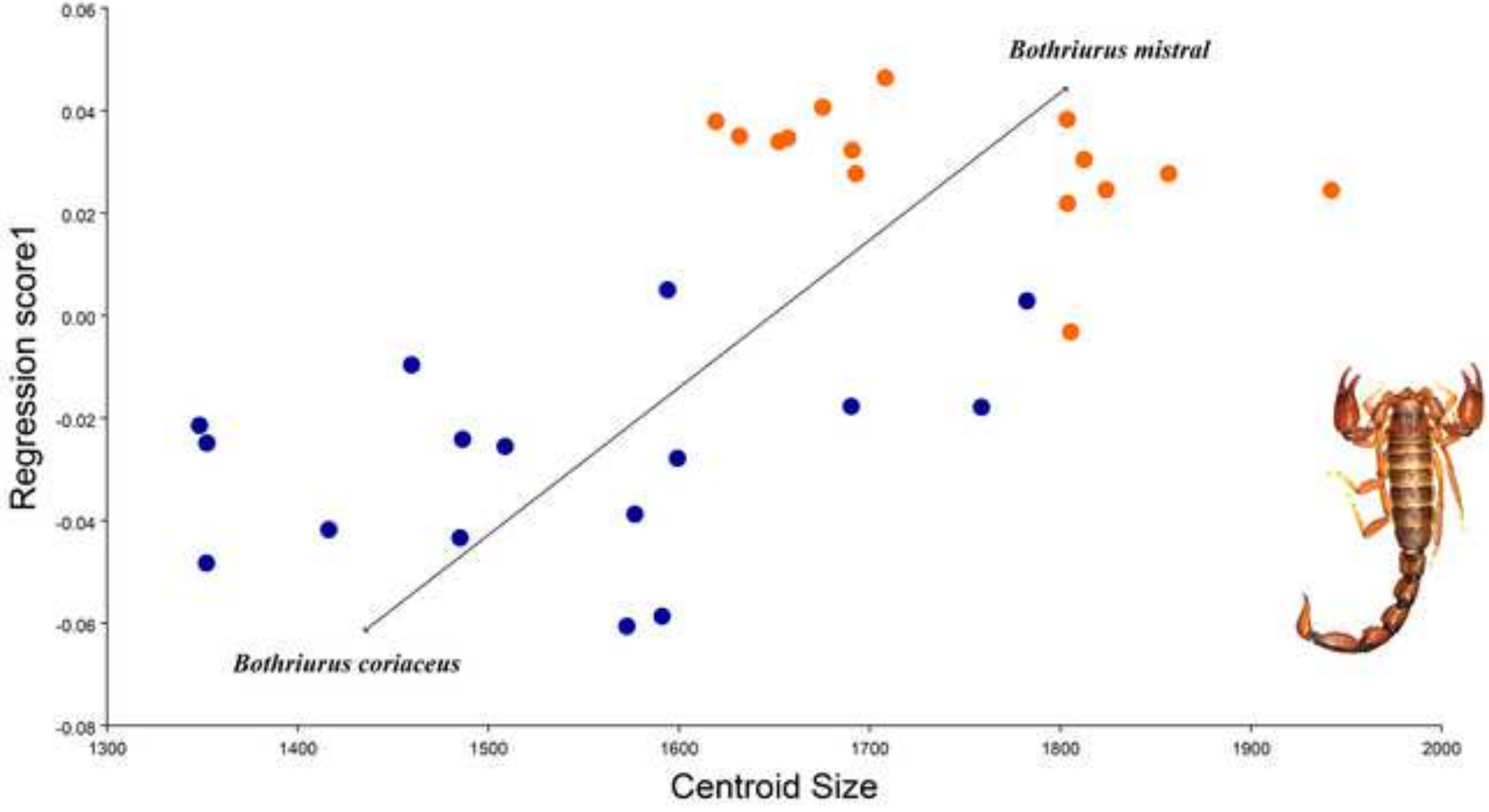

Figure 9

Click here to access/download;Figure;Fig 9-Ojanguren et al. 2022-Bothriurus mistral.tif

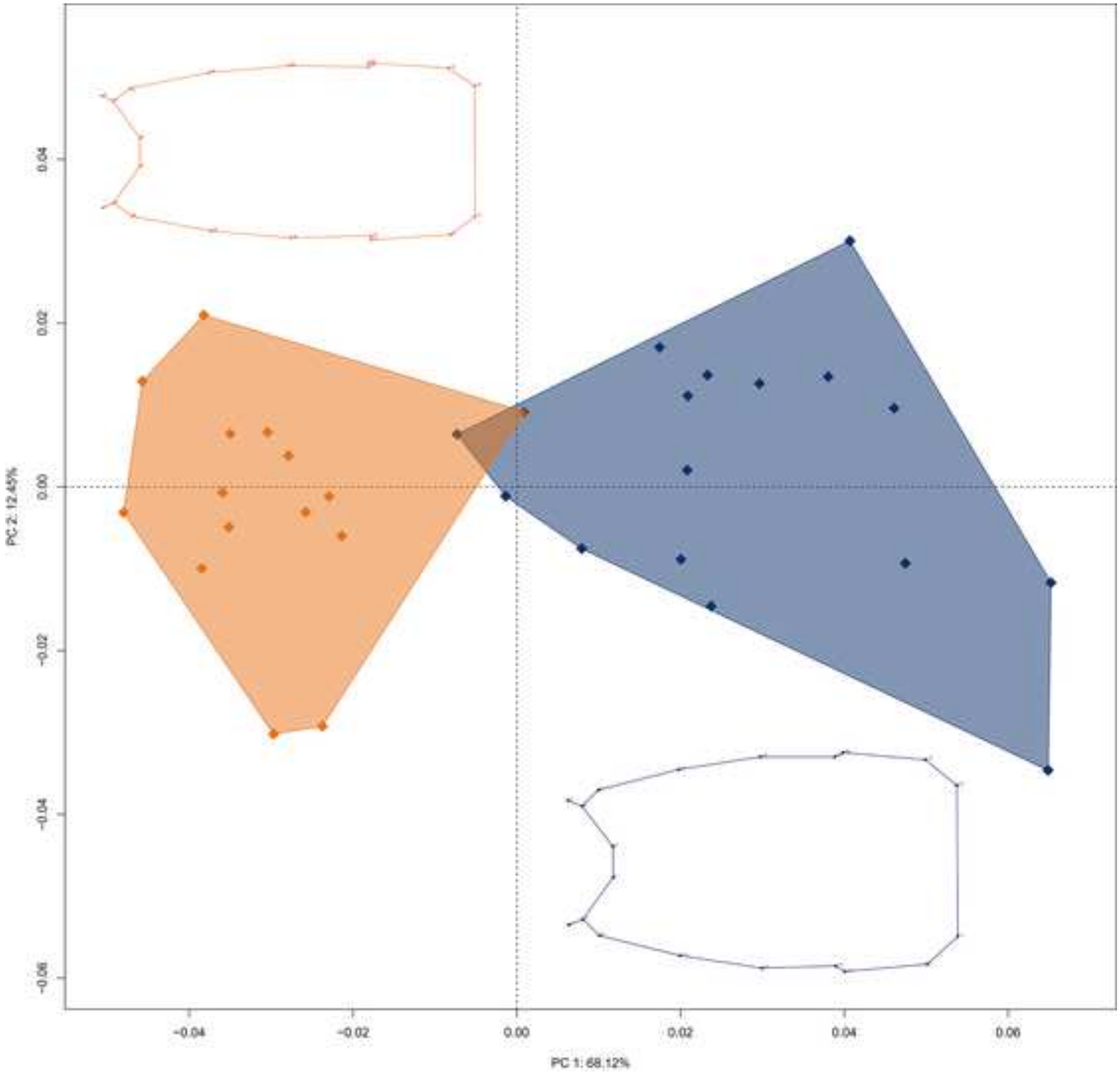

Supplement: Attachment — Submitted filename: PONE-D-22-27368-1.pdf [file pone.0281336.s001.pdf]
